# Effects of Intraoperative Epidural Steroid-Soaked Gelatin Sponge in Lumbar Spine Surgery: A Systematic Review and Meta-Analysis of Randomized Controlled Trials

**DOI:** 10.7759/cureus.71855

**Published:** 2024-10-19

**Authors:** Hong-Xiang Zheng, Wen-Wen Tsai, Pei-Hua Wu

**Affiliations:** 1 Neurosurgery, Chi-Mei Medical Center, Tainan, TWN; 2 Neurology, Chi-Mei Medical Center, Tainan, TWN; 3 Biostatistics, National Health Research Institutes, Tainan, TWN

**Keywords:** lumbar spine surgery, pain control, postoperative analgesic demand, steroid-soaked gelatin sponge, vas pain score

## Abstract

This systematic review and meta-analysis evaluated the impact of intraoperative epidural steroid-soaked gelatin sponge in lumbar spine surgery for conditions like spinal stenosis and nerve root compression. Surgery often involves removing structures to decompress the nerves. Steroids are used to alleviate inflammation and pain, with gelatin sponges serving as carriers to extend their effect. While epidural steroid injections have been effective, the combined use of gelatin sponges remains controversial.

The study systematically searched databases including PubMed, MEDLINE, and Cochrane Central Registry of Controlled Trials databases involving lumbar surgeries using steroid-soaked gelatin sponges. Seven studies with 620 patients were included, focusing on postoperative pain scores, analgesic use, hospital stay, and complications. The quality of the studies was assessed using the Cochrane Risk of Bias 2 tool.

Results showed no significant difference in pain scores between the steroid and control groups on postoperative day 1 and at three months. However, there was a trend toward reduced pain in the steroid group. Notably, patients in the steroid group required fewer analgesics, indicating a significant reduction in postoperative pain medication use. Other factors, such as surgery time, blood loss, and hospital stay, showed no statistically significant differences between the groups. The findings suggest that while the use of steroid-soaked gelatin sponges does not significantly change immediate postoperative pain levels, it may contribute to improved pain management. This approach is safe, with minimal risk of complications, and may provide prolonged, localized effects due to the slow release of steroids. Future large-scale randomized trials are needed to further validate these findings.

In conclusion, the use of steroid-soaked gelatin sponges in lumbar spine surgery is a feasible option to consider, with potential benefits in postoperative pain management and reduced analgesic consumption.

## Introduction and background

Lumbar spine nerve root compression and spinal stenosis are common causes of low back pain and sciatica [1]. Surgery is often necessary for patients with severe spinal stenosis or those who have not responded to conservative treatment. The primary goal of the surgery is to decompress and relieve the nerve by removing structures such as the ligamentum flavum or herniated disc. In addition to direct nerve compression, proinflammatory substances can also contribute to nerve irritation and subsequent pain. The intraoperative use of epidural steroids has shown promising results in reducing pain by alleviating inflammation [2]. To extend the duration and effect of steroids, a Gelatin sponge is often used as a drug carrier. Gelatin sponge is a hemostatic agent commonly employed in various surgeries and is typically absorbed within four to six weeks.

From a surgical perspective, decompression remains the primary objective. However, to optimize surgical outcomes, we aim to incorporate anti-inflammatory agents such as a steroid-soaked gelatin sponge at the surgical site toward the end of the procedure. This addition may help to further reduce postoperative pain, enhancing the overall effectiveness of the surgery. The application of a steroid-soaked gelatin sponge in the epidural space is intended to keep the steroid localized for a longer period, potentially enhancing its effect. However, while epidural steroid injections alone have demonstrated effectiveness, the efficacy of combining gelatin sponge with steroids remains unclear.

This systematic review and meta-analysis of randomized controlled trials aim to thoroughly evaluate the impact of using intraoperative epidural steroid-soaked gelatin sponge in lumbar spine surgery, focusing on its potential benefits in reducing postoperative pain, minimizing the need for analgesics, and enhancing overall surgical outcomes by prolonging the anti-inflammatory effects at the surgical site. Additionally, the review will examine any potential complications and the overall feasibility of incorporating this technique into standard surgical practice.

## Review

Method

Search Strategy

A systematic search of the PubMed, EMBASE, and Cochrane Central Registry of Controlled Trials databases was conducted according to the Preferred Reporting Items for Systematic Reviews and Meta-Analyses (PRISMA) guidelines, covering studies from inception up to August 31, 2024 [3]. Potentially eligible studies were identified by searching the PubMed, EMBASE, and Cochrane databases using the following terms: discectomy, fusion, laminectomy, lumbar surgery, steroid, anti-inflammatory, dexamethasone, methylprednisolone, Depo-Medrol, and triamcinolone. The full texts of potentially relevant studies were then retrieved and assessed.

**Figure 1:**
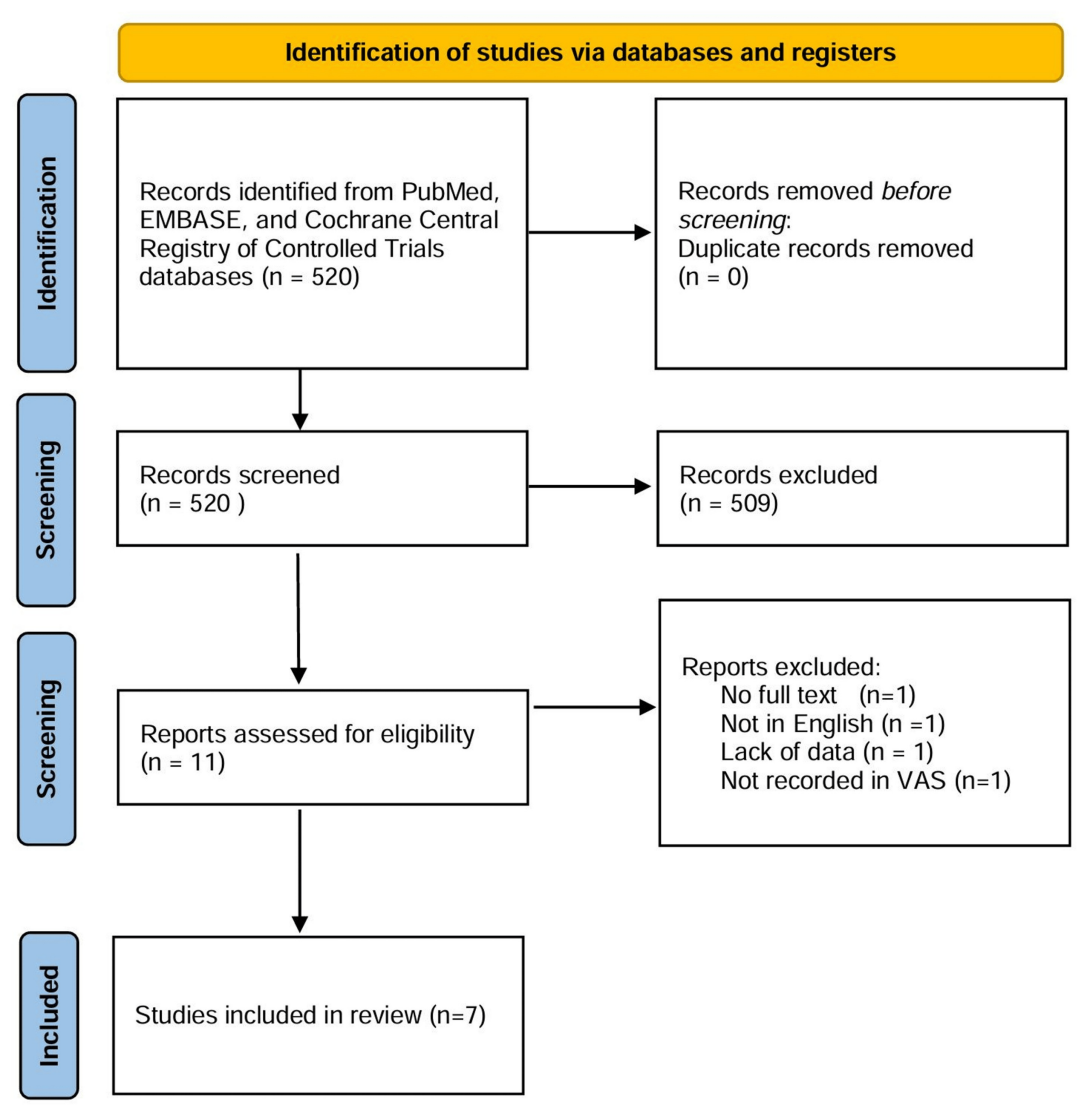
PRISMA flow diagram PRISMA, Preferred Reporting Items for Systematic Reviews and Meta-Analyses

Eligibility Criteria

The inclusion criteria were randomized controlled trials involving patients aged 18 years or older who underwent lumbar surgery with the application of a steroid-soaked gelatin sponge in the epidural space. Studies were excluded if the full text was not available, was not written in English, or was duplicated. The included studies were required to provide sufficient data on at least part of the following outcome criteria: pain assessment scores at specific time points in the postoperative period.

Data Extraction

Data were extracted from the included studies using a standardized form. Two investigators performed this process independently and then compared their results to minimize extraction errors. Any missing data were marked as not available. The following information was collected for each study: year of publication, number of patients treated with steroids, number of controls, total number of patients in the trial, dose of steroids and any additional medications administered, method of pain score assessment, all recorded postoperative pain scores for both back pain (BP) and radicular leg pain (RLP) along with their timing, postoperative analgesia consumption, duration of hospital stay, and rates of complications such as infection and recurrence of disc prolapse.

Quality Assessment

For the quality assessment, the Risk of Bias 2 tool from the Cochrane Handbook for Systematic Reviews of Interventions was used [4]. Two investigators, Hong Xiang Zheng and Wen-Wen Tsai, independently evaluated the selected studies based on five different domains, rating each criterion as "low," "some concerns," or "high" risk of bias. The levels of evidence were determined using the Oxford Centre for Evidence-Based Medicine Levels of Evidence tool (2011) (OCEBM Levels of Evidence Working Group, Durieux et al., 2011) [5].

Statistical Analysis

The analysis was performed using RevMan 5.4.1 software (The Cochrane Collaboration, London, England, UK) to calculate key metrics such as risk ratio, random effects, standard error (SE), and confidence intervals (CIs) for event-related studies. To assess heterogeneity, a multivariate random-effects meta-regression was conducted, with I² values categorized into four quartiles: low (0% to <25%), low-to-moderate (25% to <50%), moderate-to-high (50% to <75%), and high (>75%). A quality assessment summary bar graph and a funnel plot asymmetry test were utilized to quantify publication and item bias. All analyses were considered statistically significant at the 0.05 level.

Results

Study Characteristics

The literature search in the PubMed, EMBASE, and Cochrane Central Registry of Controlled Trials databases yielded 520 articles, of which seven randomized controlled trials were selected for inclusion (Figure 1). These included two studies on lumbar discectomy, two on lumbar laminectomy, two on lumbar spine interbody fusion, and one on posterolateral lumbar fusion [6-12]. A total of 620 patients were included in these studies, with 308 patients receiving an intraoperative epidural steroid-soaked gelatin sponge during lumbar surgery and 312 serving as controls. Details of the steroid mixtures and the sizes of the gelatin sponges used are provided in Table 1. Postoperative pain scores were assessed using the Visual Analog Scale (VAS), and the follow-up periods are also summarized in Table 1.

**Table 1:** Characteristics of the included studies

Author	year	Study design	Type of spine surgery	Number of patients (steroid:control)	Drug mixture in the intervention group	Gelatin sponge size	Control	VAS pain scale follow-up after surgery
Modi et al. [6]	2009	Prospective, randomized, case-control study	Single-level lumbar discectomy	57 (29:28)	40 mg methylprednisolone acetate	2.5×2.5 cm	No gelatin sponge was applied	2 weeks, 1 month, 3 months, 6 months, 1 year
Aljabi et al. [7]	2015	Prospective, randomized, case-control study	Lumbar microdiscectomy	150 (75:75)	Gelatin sponge soaked in 80 mg methylprednisolone acetate	Not mentioned	Saline-soaked gelatin sponge	At discharge, 1 week, 1 month
Kumari et al. [8]	2018	Prospective, randomized, double-blinded trial	Single-level lumbar laminectomy	50 (25:25)	Gelatin sponge soaked in 10 mL of 0.25% levobupivacaine + 2 mL of dexamethasone.	5 × 2 × 1 cm	Gelatin sponge soaked in 12 mL of 0.9% sodium chloride	1, 2, 4, 8, 12, 18, 24 hours
Haws et al. [9]	2019	Prospective, randomized, single-blind trial	Single-level minimally invasive transforaminal lumbar interbody fusion	93 (45:48)	1 ml depomedrol (80 mg)	10 cm2	1 ml saline	6 weeks, 12 weeks, 6 months
Du et al. [10]	2021	Prospective, randomized, double-blinded trial	Minimally invasive-transforaminal lumbar interbody fusion	128 (63:65)	3 mL dexamethasone injection (1 mL:5 mg) plus 2 mL vitamin B12 injection (2 mL:0.5 mg)	3 cm × 2 cm × 0.5 cm	2.5 ml of normal saline	Day 1 to day 10
Tavanaei et al. [11]	2022	Prospective, randomized, double-blinded trial	Posterolateral lumbar fusion surgery	100 (50:50)	1 ml of triamcinolone acetonide (40 mg)	Adjusted based on the epidural space	Normal saline + gelatin sponge	2, 4, 6, 12, 24, 48 hours and 4, 12 weeks
Saebi et al. [12]	2022	Prospective, randomized, triple-blinded trial	Unilateral laminectomy surgery	42(21:21)	2 cc of dexamethasone (8 mg) and bupivacaine (4 cc of 0.5% solution)	2 × 1 cm	6 cc of normal saline	3, 6, 12, 24 hours and 1, 3, and 6 months

Assessment of Item Risk of Bias of the Studies

**Figure 2:**
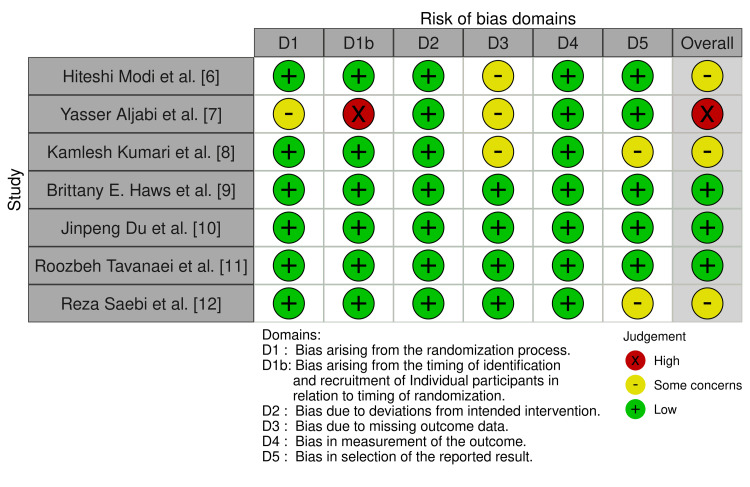
Risk-of-bias traffic light plot Sources: Modi et al., 2009 [6]; Aljabi et al., 2015 [7]; Kumari et al., 2018 [8]; Haws et al., 2019 [9]; Du et al., 2021 [10]; Tavanaei et al., 2022 [11]; Saebi et al., 2022 [12]

The risk of bias assessment was conducted using the robvis (Risk-Of-Bias VISualization) tool [13]. The results indicate that among the seven included articles, only one exhibited a high risk of bias, three showed a low risk of bias, and the remaining three raised some concerns. The identified issues involved inappropriate handling of the randomization process, the timing of participant identification and recruitment in relation to randomization, missing outcome data, and the selection of reported results. Further details are provided in Figure 2.

Meta-Analysis of the Studies' Outcome

**Figure 3:**
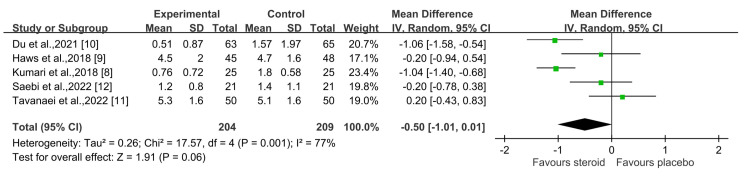
Posteoperative day 1 VAS pain score Sources: Kumari et al., 2018 [8]; Haws et al., 2019 [9]; Du et al., 2021 [10]; Tavanaei et al., 2022 [11]; Saebi et al., 2022 [12] VAS, Visual Analog Scale

This forest plot presents the outcomes of steroid use on postoperative day 1 VAS (Visual Analog Scale) scores across five studies (Figure 3) [9-12]. The analysis revealed significant heterogeneity (I² = 77%, X² = 17.57), with no statistically significant difference between the two groups at a 95% confidence interval of -0.50 (-1.01, 0.01) (P = 0.06). The variability among the included studies may be attributed to differences in study design, patient populations, or the specific steroid regimens used.

**Figure 4:**
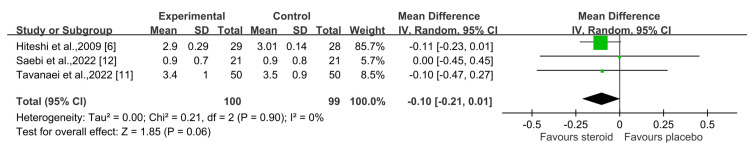
Postoperative three-month VAS pain score Sources: Modi et al., 2009 [6]; Tavanaei et al., 2022 [11]; Saebi et al., 2022 [12] VAS, Visual Analog Scale

At the postoperative three-month mark, the results showed no statistically significant difference in VAS scores between the groups (I² = 0%, X² = 0.21) with a 95% confidence interval of -0.10 (-0.21, 0.01) (P = 0.06) (Figure 4) [6,11,12].

**Figure 5:**
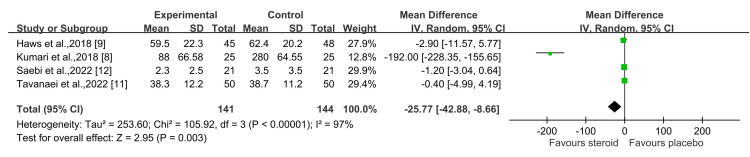
Postoperative analgesic use (mg) Sources: Kumari et al., 2018 [8]; Haws et al., 2019 [9]; Tavanaei et al., 2022 [11]; Saebi et al., 2022 [12]

Regarding postoperative analgesic use, the findings indicate that the steroid group required significantly fewer analgesic drugs compared to the control group (P = 0.003) with a 95% CI of -25.77 (-42.88, -8.66) (Figure 5) [8,9,11,12].

**Figure 6:**
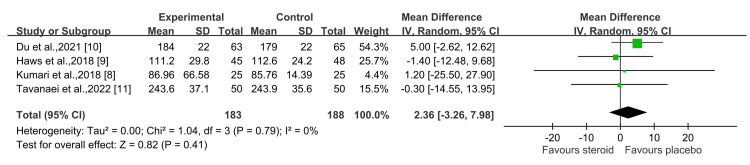
Surgery duration (minutes) Sources: Kumari et al., 2018 [8]; Haws et al., 2019 [9]; Du et al., 2021 [10]; Tavanaei et al., 2022 [11]

For surgery time, the steroid group had a longer operative duration than the control group; however, this difference was not statistically significant (P = 0.41) at a 95% CI of 2.36 (-3.26, 7.98) (Figure 6) [8-11].

**Figure 7:**
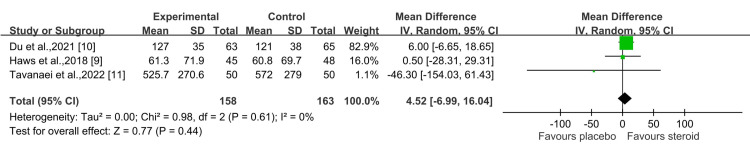
Intraoperative blood loss (ml) Sources: Haws et al., 2019 [9]; Du et al., 2021 [10]; Tavanaei et al., 2022 [11]

In terms of intraoperative blood loss, the steroid group had a higher volume compared to the control group, though this difference was also not statistically significant (P = 0.44) at a 95% CI of 4.52 (-6.99, 16.04) (Figure 7) [9-11].

**Figure 8:**
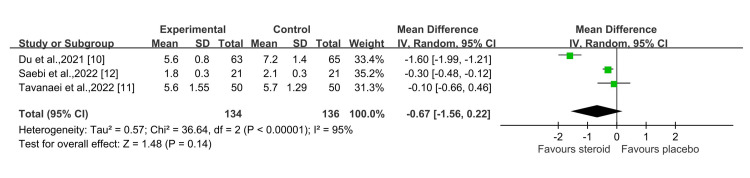
Hospital stay (days) Sources: Du et al., 2021 [10]; Tavanaei et al., 2022 [11]; Saebi et al., 2022 [12]

Regarding hospital stay, the steroid group had a shorter duration of stay than the control group; however, this difference was not statistically significant (P = 0.14) at a 95% CI of -0.67 (-1.56, 0.22) (Figure 8) [10-12].

Discussion

This systematic review and meta-analysis aimed to assess the effects of using an intraoperative steroid-soaked gelatin sponge in lumbar spine surgery. The findings indicate that there is no significant difference in pain scores between the steroid and non-steroid groups on postoperative day 1 and at three months, and neither group showed a significant reduction in pain compared to the control. From our perspective, although steroids can alleviate pain caused by inflamed nerves, the surgery itself already provides a significant level of pain relief. As a result, the marginal benefit of steroids in further reducing pain diminishes, leading to a positive but not statistically significant effect in the analysis. Regarding postoperative analgesic use, the results demonstrate that the steroid group required significantly fewer analgesics than the control group, with statistical significance (P=0.003) at a 95% CI of -25.77 (-42.88, -8.66). The heterogeneity in the analysis primarily stems from the study by Kumari et al., 2018 [8]. However, when this study was excluded from the analysis, the statistical significance disappeared, although the use of steroids still demonstrated a positive effect, without statistical significance (P=0.18) at a 95% CI of -1.16 (-2.83, 0.52). Regarding operative time and intraoperative blood loss, patients who received steroids were observed to have slightly more blood loss and longer operative times; however, these differences were not statistically significant. Typically, the procedure of placing an intraoperative epidural steroid-soaked gelatin sponge is performed as the final step of the surgery, and the preparation and placement usually take no more than five minutes. Therefore, in our view, the blood loss is likely not directly related to the placement of the intraoperative epidural steroid-soaked gelatin sponge. However, it may slightly extend the overall operative time. In our experience, this additional time is acceptable.

Compared to a simple steroid epidural injection, the advantage of using a steroid-soaked gelatin sponge lies in its slow release of medication as the gelatin sponge is gradually absorbed. Additionally, the sponge can retain the steroid near the inflamed tissue, preventing it from spreading to other areas. Therefore, this method may provide a more prolonged and targeted local effect. Glucocorticoids are known to have several detrimental effects on bone health, primarily by inhibiting osteoblastogenesis, which is the formation of new osteoblasts, and by promoting the apoptosis, or programmed cell death, of both osteoblasts and osteocytes [14]. Osteoblasts are essential for bone formation and repair, while osteocytes play a crucial role in maintaining bone tissue. When glucocorticoids interfere with these cells, it results in decreased bone formation and increased bone fragility. This mechanism raises concerns in lumbar fusion surgeries, where the use of steroids could potentially lead to complications such as pseudoarthrosis. Pseudoarthrosis is the failure of bone fusion following surgery, resulting in the formation of a false joint, which can lead to instability, pain, and functional impairment. Pseudoarthrosis can be a significant issue in spinal fusion procedures, potentially affecting the long-term success and stability of the surgery. Although there is evidence from a study indicating that local steroid injection in 1-level or 2-level lumbar fusion surgeries does not lead to postoperative pseudoarthrosis, this finding does not entirely mitigate the concern [15]. The pharmacological mechanisms of steroids, which include their impact on bone remodeling, cellular turnover, and healing processes, still raise apprehensions about their overall safety in this context. Therefore, while some evidence suggests that steroid use may be relatively safe, the potential risk of affecting bone healing remains a point of caution that needs to be carefully considered. The use of a steroid-soaked gelatin sponge can potentially limit the spread of the medication, reducing the likelihood of it reaching the disc space and affecting adjacent tissues. This localized application not only provides targeted anti-inflammatory effects but also minimizes the risk of unintended steroid distribution. In the literature review conducted for this study, there were no reported complications associated with the use of steroids or any adverse effects related to the mass or placement of the gelatin sponge. This suggests that, when applied appropriately, the gelatin sponge serves as a safe and effective method for delivering steroids during surgery. In addition to pseudoarthrosis, infection is another complication that requires careful consideration when using steroids. A systematic review and meta-analysis indicated a trend toward an increased risk of infection associated with the intraoperative use of epidural steroids, although this finding did not reach statistical significance [2]. In our current systematic review, we also did not observe any reports of infection, suggesting that the use of a steroid-soaked gelatin sponge appears to be a safe procedure.

This study has several limitations. One notable concern is related to the gelatin sponge medication. Although the drug mixture in each study consistently included steroids, the specific composition and concentration of steroids varied across the studies. This inconsistency in the formulation may have introduced a level of bias into the results, potentially affecting the overall interpretation of the effectiveness and safety of the steroid-soaked gelatin sponge. Additionally, differences in surgical techniques, dosage, and patient selection criteria across studies could further contribute to variability in outcomes. Additionally, the articles included in this review did not specifically address infection and pseudoarthrosis rates, leaving an important aspect unexamined. Future study designs should consider incorporating infection and pseudoarthrosis rates assessments to provide a more comprehensive evaluation of the safety profile of steroid use when spine fusion surgery is involved particularly. We also hope that larger-scale randomized controlled trials will be conducted to explore this area in greater depth, providing more definitive insights into the potential risks and benefits, without concerns of publication bias influencing the dissemination of findings.

## Conclusions

The study demonstrated that the use of a steroid-soaked gelatin sponge in lumbar spine surgery showed no significant difference in pain levels on postoperative day 1 and at three months. However, the study observed a reduction in the need for analgesics, indicating a potential benefit in managing postoperative pain. Given that this procedure carries minimal risk of complications, the use of a steroid-soaked gelatin sponge in lumbar spine surgery remains a feasible and potentially beneficial option for pain management.
